# TLR13 contributes to skeletal muscle atrophy by increasing insulin resistance in chronic kidney disease

**DOI:** 10.1111/cpr.13181

**Published:** 2022-01-28

**Authors:** Lijie Gu, Zhifang Wang, Yueyue Zhang, Nan Zhu, Jiayong Li, Man Yang, Ling Wang, Shu Rong

**Affiliations:** ^1^ Department of Nephrology Shanghai General Hospital Shanghai Jiao Tong University School of Medicine Shanghai China; ^2^ Department of Respiration Yangpu Hospital School of Medicine Tongji University Shanghai China; ^3^ Clinical Laboratory Medicine Center Shanghai General Hospital Shanghai Jiao Tong University School of Medicine Shanghai China

**Keywords:** chronic kidney disease, insulin resistance, muscle atrophy, TLR13

## Abstract

**Objectives:**

Insulin resistance in chronic kidney disease (CKD) stimulates muscle wasting, but the molecular processes behind the resistance are undetermined. However, inflammation in skeletal muscle is implicated in the pathogenesis of insulin resistance and cachexia. Toll‐like receptors (TLRs) are known to regulate local innate immune responses, and microarray data have shown that *Tlr13* is upregulated in the muscles of mice with CKD, but the relevance is unknown.

**Materials and Methods:**

We performed in vitro experiments in C2C12 myotubes and constructed a CKD murine model using subtotal nephrectomy to conduct experiments in vivo.

**Results:**

*Tlr13* expression was stimulated in C2C12 myotubes treated with uremic serum. The expression of *Tlr13* was also upregulated in the tibialis anterior muscles of mice with CKD. *Tlr13* knockdown with siRNAs in skeletal muscle cells decreased insulin resistance despite the inclusion of uremic serum. This led to increased levels of p‐AKT and suppression of protein degradation. Using immunofluorescence staining and coimmunoprecipitation assay, we found that TLR13 recruits IRF3, which activates *Irf3* expression, resulting in decreased AKT activity. Moreover, insulin resistance and proteolysis are re‐induced by *Irf3* overexpression under *Tlr13* deletion.

**Conclusions:**

Our results indicate that TLR13 is involved in CKD‐mediated insulin resistance in muscle. In catabolic conditions where insulin signaling is impaired, targeting TLR13 may improve insulin sensitivity and prevent muscle atrophy.

## Graphical abstract

Increased levels of TLR13 promote skeletal muscle atrophy and that TLR13 interacts with IRF3, thereby impairing intracellular insulin signaling. The interaction between TLR13 and IRF3 expression resulted in decreased AKT activity, whereas IRF3 knockdown prevented TLR13‐induced insulin resistance and increased p‐AKT. TLR13 is part of a novel mechanism of CKD‐mediated insulin resistance in muscle. In addition, we propose that the ETS2 protein may activate the TLR13 promoter and promote the expression of TLR13 at the transcriptional level.

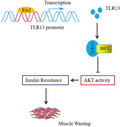

## 1 INTRODUCTION

Muscle atrophy attributed to chronic kidney disease (CKD) is believed to be a consequence of dysfunction in the signaling pathways that regulate protein degradation.^1^, ^2^, ^3^ CKD‐induced muscle wasting is difficult to treat because protein turnover is quite low and even a small unbalance can result in significant muscle loss, which results in a far greater risk of mortality.^2^, ^4^ The potential etiology of muscle atrophy in CKD includes metabolic acidosis, inflammation, and insulin resistance.^5^, ^6^, ^7^, ^8^ Insulin resistance was first noted in patients with metabolic syndrome who later went on to develop CKD and muscle wasting.^9^ However, insulin resistance has also been observed in patients who do not have metabolic syndrome and evidence indicates that altered muscle mitochondrial function may be involved but the exact mechanism is unclear.^10^

Increasing evidence implicates proinflammatory factors, such as interleukin (IL)‐6, tumor necrosis factor (TNF)‐α, and Toll‐like receptors (TLRs) in accelerating the degradation of proteins and insulin resistance in CKD.^11^, ^12^, ^13^ TLRs activate the innate immune system in response to infection but can also detect danger‐associated molecular patterns related to tissue damage.^14^ The overexpression of TLR2 and TLR4 can result in the upregulation of IL‐6, which consequently leads to increases in muscle atrophy.^15^ Several TLRs have been associated with CKD, including TLR13, which is mostly associated with antiviral activity and can also activate the expression of IL‐6.^16^ Microarray analysis has indicated that TLR13 is upregulated in a mouse model of CKD and, therefore, may contribute to the overactivation of the immune system that accompanies insulin resistance.^17^

E26 transformation‐specific proto‐oncogene 2 (ETS2) is a transcription factor belonging to the ETS family that may be involved in the regulation of TLRs. ETS2 is thought to interact with TLR13, and its overexpression leads to a higher expression of TLR13, whereas the activation of TLR2 and TLR3 inhibits TLR13 expression.^18^ In addition, NF‐κB was found to inhibit the expression of *Tlr13* transcription whereas interferon‐β can activate *Tlr13* transcription.^18^ More recently, ETS2 was found to suppress the induction of IL‐6 expression by TLRs through mediating the histone deacetylation of the IL‐6 promoter.^19^

Toll‐like receptors are believed to control the interferon regulatory factor (IRF) family of transcription factors.^20^ In the past, TLRs were thought to induce interferon responses by activating IRF3 and IRF7 to modify the immune response but more recent evidence suggests that TLRs activate specific IRFs depending on the TLR ligand.^21^ Activated TLR signaling could recruit and phosphorylate IRF3 by complexing with cofactors, such as TRIF and TBK1. Therefore, we hypothesized that the signal cascade initiated by TLR13 might be the result of the recruitment of IRF3 with TLR13. Kumari et al.^22^ found high levels of IRF3 in adipocytes during obesity and high expression of TLR3 and TLR4, which are upstream activators of IRF3. The activation of IRF3 is believed to induce insulin resistance, whereas its inhibition prevents insulin resistance. Moreover, IRF3‐deficient mice were found to have an increased sensitivity to insulin and reduce systemic inflammation.

AKT (also known as protein kinase B) signaling plays a central role in insulin resistance and skeletal muscle atrophy.^23^, ^24^ The Ser‐473 residue in AKT is believed to regulate its activity; increased phosphorylation indicates activation and leads to insulin sensitivity, whereas decreased phosphorylation leads to insulin resistance. AKT is believed to regulate the insulin signaling pathway mutually with glycogen synthase kinase (GSK)‐3β.^25^, ^26^ Under normal conditions, AKT inhibits GSK‐3β activity to increase insulin sensitivity, whereas under CKD, the inhibition of AKT activation by uremic toxins leads to insulin resistance by activating GSK‐3β.^25^ AKT pathways are believed to interact with IRF3 during hyperglycemia and influence levels of inflammatory mediators, but the exact mechanism is unclear.^27^, ^28^

Through STRING predictive analysis, we found that TLR13 can interact with IRF3. Our results suggest that TLR13 recruits and phosphorylates IRF3 to inhibit AKT activity and impaired insulin signaling, thereby mediating the occurrence of CKD skeletal muscle atrophy.

## 2 MATERIALS AND METHODS

### 2.1 Cell culture and treatments

A mouse skeletal muscle cell line C2C12 was propagated as described previously.^13^ Cells were incubated at 37°C in low‐glucose Dulbecco's modified Eagle's medium (DMEM, Sigma‐Aldrich) with 2 mmol L‐glutamine, 100 U/ml penicillin‐streptomycin, and 5% FBS. Myoblasts at 90% confluence were differentiated into myotubes by adding 2% horse serum to DMEM. For evaluating TLR13 induction, myotubes were incubated with 15% normal or uremic serum for 24 h.

TLR13 (NM_205820.1) siRNA (siTLR13), ETS2 siRNA (siETS2), and recombinant adenovirus expressing *Irf3* were purchased from GE Dharmacon and transfected into cells using Lipofectamine 2000. The sequences of the siTLR13 were: siTLR13: 5′‐UACAAUUGUUCAUCUUUGCUA‐3′ and siETS2: 5′‐UCCAAAGUCAUUCAUCGCG‐3′.

Adenovirus expressing *IRF3* was constructed by cloning *Irf3* cDNA into a pAdTrack‐CMV plasmid using the AdEasy System (Clontech Laboratories). The transfected cells were differentiated into myotubes and placed in serum‐free medium before being treated for 24 h with 15% uremic serum from subtotal nephrectomy mice or normal serum from sham mice.

### 2.2 Uremic serum

Blood was drawn at euthanasia via retro‐orbital puncture. BUN and creatinine were assayed in CKD mice. Blood was collected in Vacutainer tubes, and serum was obtained by centrifuging clotted blood at 1100 g for 10 min at room temperature. To minimize minor differences between CKD mice, all serum samples were pooled for the experiments. Serum samples were frozen at −20°C until analysis.

### 2.3 Cell viability measurements

For investigating the effect of uremic serum on cell viability, C2C12 cells (6 × 10^3^ cells/well) were seeded in 96‐well plates and differentiated into myotubes. Normal or uremic serum was added, and cells were incubated for 24, 48, and 72 h. Cell viability was measured using a cell counting kit‐8 (Beyotime) following the manufacturer’s instructions.

### 2.4 RT‐qPCR

Total RNA was extracted from cells using RNAeasy (Qiagen, Hilden, Germany), and cDNAs were synthesized using a cDNA Synthesis Kit (Sigma‐Aldrich). Real‐time PCR was performed with SYBR Premix Taq II (Takara) and a CFX96 Real‐Time PCR Detection System with the following primers: TLR13, forward: TGTCTGCTCTGGTGGACTTG, reverse: GAGGAGTGA‌AGGCGTCTTTG; atrogin‐1, forward: GAGGCAGATTCGCAAGCGTTTGAT, reverse: TCCAGGAGAGA‌ATGTGGCAGTGTT; MuRF‐1, forward: AGTGTCCATG‌TCTGGAGGTCGTTT, reverse: ACTGGAGCACTCCTGCTTGTAGAT.

The relative expression of target genes was normalized to that of the housekeeping β‐actin gene using the $2^{‐\Delta \Delta C_{T}}$ method.

### 2.5 Western blot analysis and immunoprecipitation

Proteins were extracted from tibialis anterior (TA) muscles or cultured C2C12 myotubes using RIPA buffer (Thermo Fisher Scientific). Protein concentrations were measured by using a Bradford Protein Assay Kit (Bio‐Rad). About 30 mg proteins were separated by SDS‐PAGE and transferred to nitrocellulose membranes. Membranes were incubated with primary antibodies overnight at 4°C following the manufacturer’s recommendations. The following primary antibodies were used for western blot analyses: TLR13 (Imgenex; ABU009), Ets2 (Abcam; ab219948), phospho‐IRF‐3 (Ser386) (Cell Signaling Technology; #37829), IRF3 (Cell Signaling Technology, #4302), AKT (Cell Signaling Technology, #4691), phospho‐AKT (Ser473) (Cell Signaling Technology, #4060), atrogin‐1 (Abcam, ab168372), and MuRF‐1 (Abcam, ab183094). Primary antibodies were counter‐probed with secondary horseradish peroxidase antibodies (Sigma‐Aldrich) for 1 h at room temperature. Immunoreactive bands were visualized using enhanced chemiluminescence, and images were captured and analyzed using Image Lab software (Bio‐Rad).

To perform immunoprecipitation (IP), 4 μg anti‐TLR13 or anti‐IRF3 antibodies were added to the C2C12 lysates and incubated overnight at 4°C with phosphatase and protease inhibitors. Protein A/G Plus Beads (Thermo Fisher Scientific) were added, and the sample was incubated for a further 2 h with agitation. Proteins were collected by centrifugation at 1000 *g* for 5 min at room temperature and then analyzed by western blotting.

### 2.6 Morphology to assess differentiation and myotube hypertrophy/atrophy

The myotube area was analyzed using a live imaging light microscope at ×10 or ×20 magnification (AF600 Modular System). The myotube number, diameter, and area were assessed in each experimental condition at each time point in six images per well, and data were analyzed using ImageJ software (National Institutes of Health, Bethesda, MD, USA).

### 2.7 Protein synthesis and degradation using isotopes

Protein synthesis and degradation in C2C12 myotubes were measured by adding isotopes to media as described previously.^29^ Briefly, after incubating myotubes in media containing L‐[3, 5–3H] tyrosine (5 mCi/ml; PerkinElmer) for 24 h, the L‐3 H‐tyrosine in cell proteins was measured using a liquid scintillation counter and protein concentration was measured using a Pierce BCA Protein Assay (Thermo Fisher Scientific). Radioactivity released from total cell proteins was plotted as a percentage of total [3H] tyrosine. A linear slope between 24 and 36 h was used to calculate the rate of protein degradation.

### 2.8 Determination of glucose consumption and glucose uptake in C2C12 cells

To determine glucose consumption and uptake in cells, a previously published protocol was followed.^30^ Briefly, glucose consumption was determined as the difference between the initial and residual glucose concentrations in the culture medium. A Glucose Uptake Assay Kit (Abcam) was used to measure glucose uptake in cells. C2C12 cells were incubated with or without 100 nM insulin for 10 min. Cells were then incubated with the addition of 2‐[N‐(7‐nitrobenz‐2‐oxa‐1,3‐diazol‐4‐yl) amino]‐2‐deoxy‐D‐glucose (Invitrogen) for 30 min. Fluorescence intensity was measured at 485/535 nm (excitation/emission) using a fluorescence microplate reader.

### 2.9 Luciferase reporter assays

To determine whether ETS2 interacted with TLR13, a Dual‐Luciferase Reporter Assay System (Promega Corporation) was used as described previously.^31^ The 1‐kb promoters (upstream of the translation initiation site ATG) of TLR13 were cloned using PrimeStar DNA polymerase‐mediated high‐fidelity PCR (Takara). Reporter plasmid (200 ng) and the Renilla luciferase plasmid (10 ng; internal control) were cotransfected into HEK293T cells using Lipofectamine 2000. Relative luciferase activity was used to validate whether ETS2 regulates the expression of TLR13 by binding to the promoter.

### 2.10 Animal models and electroporation

Male C57BL/6 mice (8 weeks old) underwent subtotal nephrectomy to induce CKD by a method described previously.^32^ Briefly, mice were anesthetized with ketamine (125 mg/kg body wt) and xylazine (6.4 mg/kg body wt). The left kidney was decapsulated, and approximately three‐quarters was removed. During the recovery, mice received buprenorphine (0.1–2.5 mg/kg body wt). A week later, the right kidney was removed. Sham‐operated control mice were pair‐fed with CKD mice using the same diets (40% protein chow). CKD was confirmed by serum creatinine (average 2.2 ± 0.3 mg/dl in CKD groups) and BUN (average 82.1 ± 6.3 mg/dl in CKD groups). Serum creatinine level was determined using a creatinine kit (Cayman Chemicals). The TA muscles of mice with TLR13 silenced were injected with 0.01 nmol of SMARTpool siRNA targeting TLR13 (GE Dharmacon) in 20 ml of phosphate‐buffered saline; control mice received the same quantity of scrambled siRNA.

### 2.11 Myofiber size measurement

To assess differences in cross‐sectional areas of myofibers, frozen TA muscle sections (5 μm) fixed with 4% formaldehyde were blocked for 20 min. The sections were then incubated with anti‐dystrophin antibody (1:2000; Santa Cruz Biotechnology) at 4°C overnight. After washing with PBS, the samples were incubated with a secondary antibody (1:600; Thermo Fisher Scientific) for 30 min at room temperature in the dark. At least 1000 myofibers in each section were examined using NIS‐Elements software (Nikon).

### 2.12 Glucose and insulin tolerance tests

To measure glucose tolerance, mice were fasted for 16 h, and then blood was obtained from the tail vein to measure glucose levels using a Truetrack Glucometer (Nipro Diagnostics). Mice were then injected intraperitoneally with 1.5 mg glucose, and blood glucose was measured again at 30‐min intervals for 2 h. To measure insulin intolerance, blood glucose was first measured in mice that were fasted for 4 h. Subsequently, the mice were injected intraperitoneally with insulin (0.375 mU/g body weight) and blood glucose was measured again 30 and 60 min later.

### 2.13 Immunofluorescence staining

Sections of TA muscle tissue and C2C12 myotubes were stained using the following antibodies: TLR13 (Imgenex, ABU009), phospho‐AKT (Ser473) (Cell Signaling Technology, #4060), and phospho‐IRF‐3 (Ser386) (Cell Signaling Technology, #37829).

### 2.14 Statistical analysis

Data are shown as the mean ± standard error of the mean (SEM). Each experiment was repeated at least three times. Student’s *t*‐test or one‐way ANOVA were used for statistical analysis using Prism 6 (GraphPad Software). *p* < 0.05 was considered statistically significant.

## 3 RESULTS

### 3.1 TLR13 expression is elevated in the muscle of mice with CKD

**FIGURE 1:**
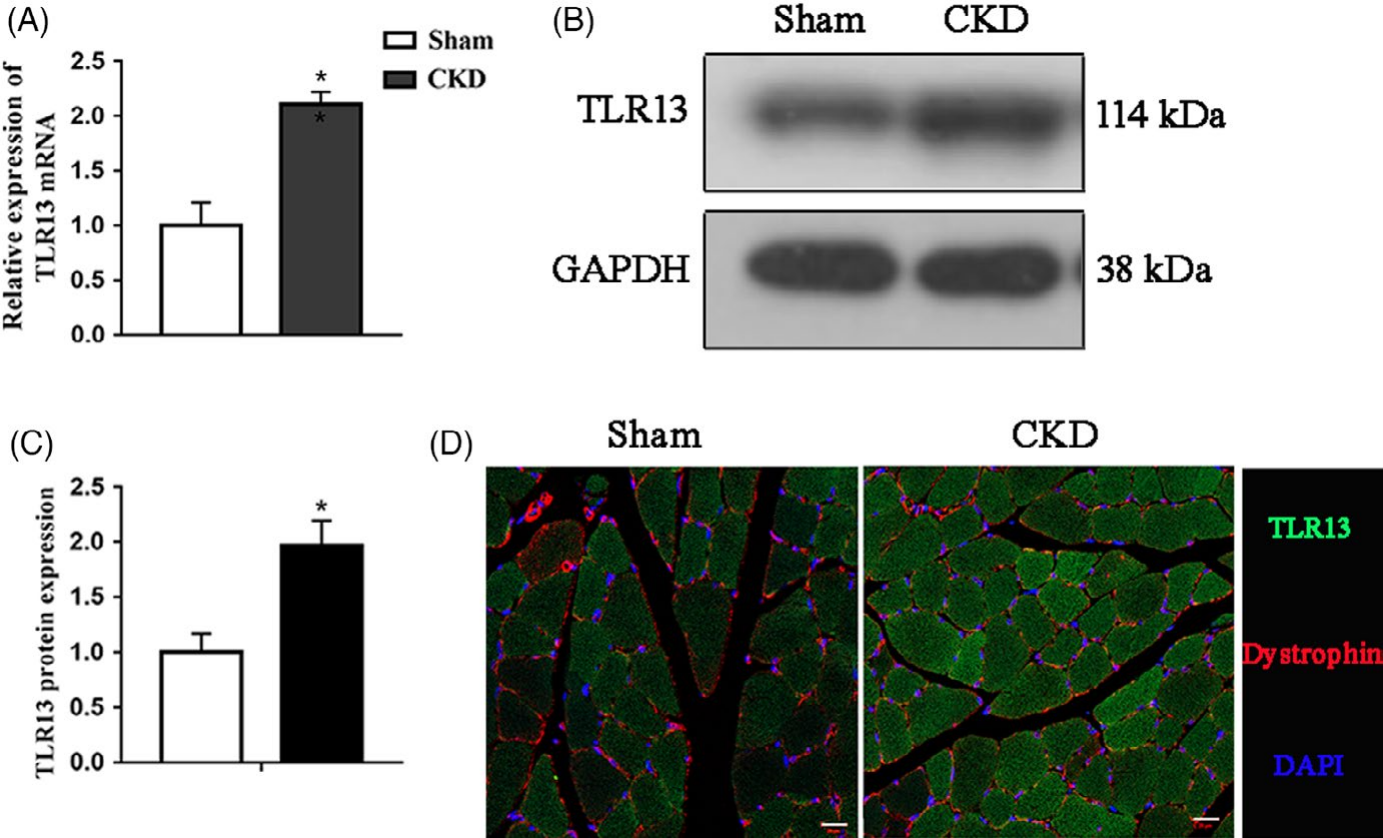
TLR13 was increased in the skeletal muscle of CKD mice. (A–C) TA muscles of CKD and pair‐fed control mice were analyzed by (A) RT‐PCR and (B, C) western blotting (*p < 0.05). (D) Immunofluorescence staining of TLR13 in TA muscle (scale bar = 20 μm)

To determine the role of TLR13 in muscle wasting, we first examined its expression levels in the skeletal muscle of a CKD mouse model. The expression levels of *Tlr13* mRNA were higher in the TA muscles of the CKD mouse model than in the sham control (Figure 1A). CKD also increased the levels of the TLR13 protein (Figure 1B,C). Immunofluorescence clearly shows the overexpression of TLR13 on the surface of muscle cells (Figure 1D). These results confirm that TLR13 is active during CKD.

### 3.2 TLR13 influenced cell viability and morphology in uremic serum‐challenged differentiated C2C12 cells

**FIGURE 2:**
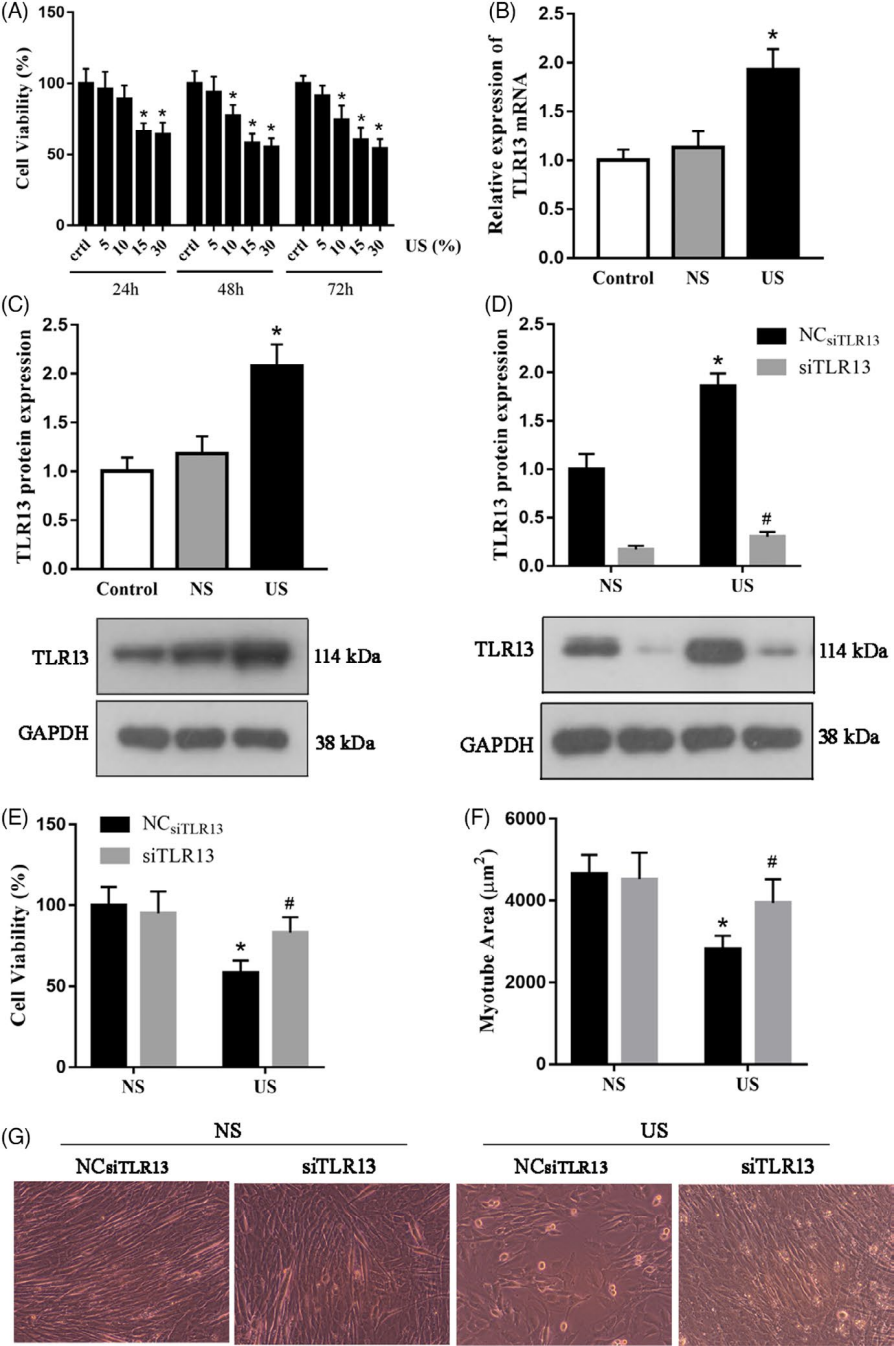
Effect of uremic serum on cell viability and morphology with or without siTLR13 during the differentiation. (A) Viability of differentiated C2C12 cells in response to different doses of uremic serum for 24, 48, and 72 h, respectively, measured by cell counting kit‐8 (mean ± SEM; *n* = 3, *p < 0.05 vs. control). (B, C) The effect of normal serum (NS) and uremic serum (US) on TLR13 mRNA and protein in C2C12 myotubes. Cells were incubated with 15% serum for 24 h. TLR13 mRNA expression was determined by real‐time PCR and levels of protein by western blot analysis (mean ± SEM; *n* = 3, *p < 0.05 vs. normal serum). (D) Gene silencing was used as an independent method to examine the role of US on TLR13 regulation. C2C12 were transfected with 60 nM siRNA NC and TLR13‐specific siRNA, and the respective protein was evaluated after 24 h. TLR13 siRNA decreased TLR13 protein in C2C12 myotubes (mean ± SEM; *n* = 3, *p < 0.05 vs. normal serum, ^#^ *p* < 0.05 vs. NC_siTLR13_+US). (E) Cell viability after the downregulation of TLR13 in 15% uremic serum‐treated C2C12 myotubes measured by cell counting kit‐8 (mean ± SEM; *n* = 3, *p < 0.05 vs. normal serum, ^#^ *p* < 0.05 vs. NC_siTLR13_+US). (F) Area of myotubes in differentiated C2C12 cells (*n* = 6 myotubes/well and three wells per treatment group, *p < 0.05 vs. normal serum, ^#^ *p* < 0.05 vs. NC_siTLR13_+US). (G) Myotube morphology in differentiating C2C12 cells transfected with or without siTLR13 and treated with 15% uremic serum for 24 h

To establish the influence of TLR13 in CKD, we challenged differentiated C2C12 cells with uremic serum. Uremic serum had a concentration‐dependent effect on cell viability (Figure 2A). A concentration of 15% or over was able to significantly reduce cell viability after 24 h. At 48 h, a uremic serum concentration of only 10% was enough to significantly reduce cell viability. The mRNA expression and protein levels of TLR13 were significantly higher in C2C12 myotubes that had been challenged with uremic serum (Figure 2B,C). After inhibiting the expression of TLR13 (Figure 2D), we found that viability improved and the area of myotubes increased in cells challenged with uremic serum compared with the control (Figure 2E–G). These results indicate that the expression of TLR13 in CKD may have a detrimental effect on the viability of cells and differentiation.

### 3.3 Knockdown of TLR13 blunts proteolysis stimulated by uremic serum in C2C12 cells

**FIGURE 3:**
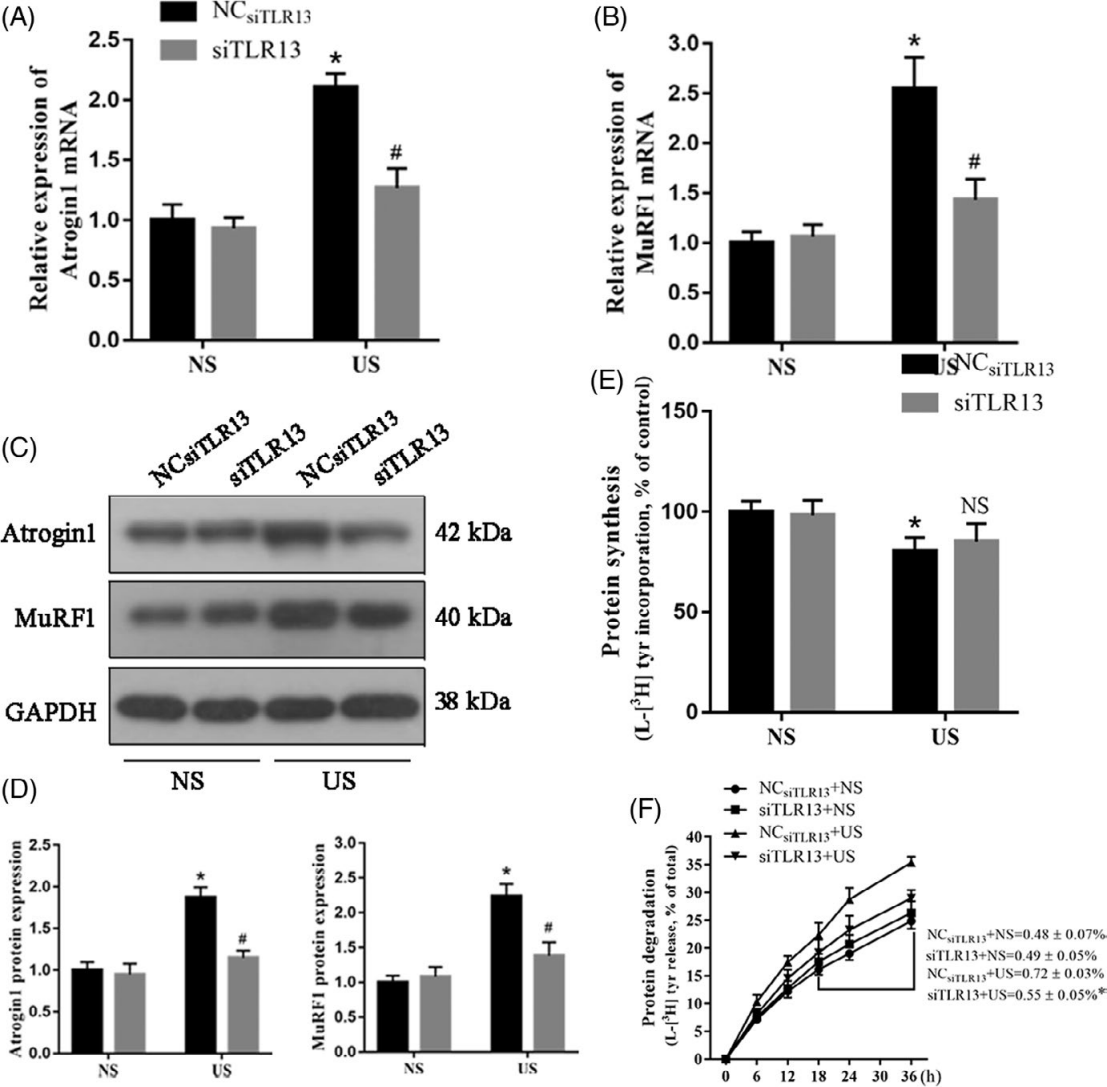
Effect of uremic serum (US) on proteolysis in C2C12 myotubes with TLR13 silenced. C2C12 myotubes with no knockdown and transfected with TLR13 siRNA were exposed for 24 h to 15% sham mouse serum (normal serum, NS) or 15% CKD mouse serum (uremic serum, US). (A, B) The mRNA level of muscle atrophic factors (atrogin‐1 and MuRF‐1) (mean ± SEM; *n* = 3, *p < 0.05 vs. normal serum, ^#^ *p* < 0.05 vs. NC_siTLR13_+US). (C, D) The protein level of muscle atrophic factors (atrogin‐1 and MuRF‐1) with a representative blot (mean ± SEM; *n* = 3, *p < 0.05 vs. normal serum, ^#^ *p* < 0.05 vs. NC_siTLR13_+US). (E) The rate of protein synthesis after the downregulation of TLR13 in US‐treated C2C12 myotubes. The rate of protein synthesis was measured from the incorporation of L‐[(3,5)‐3H] tyrosine into cellular proteins (mean ± SEM; *n* = 6, *p < 0.05 vs. normal serum, NS vs. NC_siTLR13_+US). (F) The rate of protein degradation after the downregulation of TLR13 in US‐treated C2C12 myotubes. Protein degradation was measured by the L‐[(3,5)‐3H]‐tyrosine released into media and plotted as a percentage of total L‐[(3,5)‐3H]‐tyrosine incorporated into cell proteins. The rates of proteolysis were calculated from the linear slopes between 18 and 36 h (mean ± SEM; *n* = 6, *p < 0.05 vs. NC_siTLR13_+US)

To investigate whether TLR13 may influence characteristics associated with muscle wasting, we measured the expression levels of muscle atrophic factors (atrogin‐1 and MuRF‐1) in C2C12 myotubes with TLR13 expressed or knocked down. When C2C12 myotubes were challenged with uremic serum, both atrogin‐1 and MuRF‐1 were expressed at significantly lower levels when TLR13 was knocked down (Figure 3A–D). As anticipated, levels of atrogin‐1 and MuRF‐1 were lower in cells treated with normal serum. We next estimated the rate of protein synthesis by the incorporation of L‐[(3,5)‐3H] tyrosine into cellular proteins. The application of uremic serum reduced the level of protein synthesis in C2C12 myotubes, but silencing TLR13 led to the same level of protein synthesis as in control cells treated with normal serum (Figure 3E). Protein degradation was measured by the release of L‐[(3,5)‐3H]‐tyrosine into media related to its incorporation into total cell proteins. Protein degradation was highest in cells treated with uremic serum and with TLR13 expressed (Figure 3F). Silencing *Tlr13* reduced the level of protein degradation. Overall, these findings suggest that TLR13 can influence the level of protein synthesis and proteolysis in CKD.

### 3.4 TLR13 mediates insulin resistance in response to uremic serum in differentiated muscle cells

**FIGURE 4:**
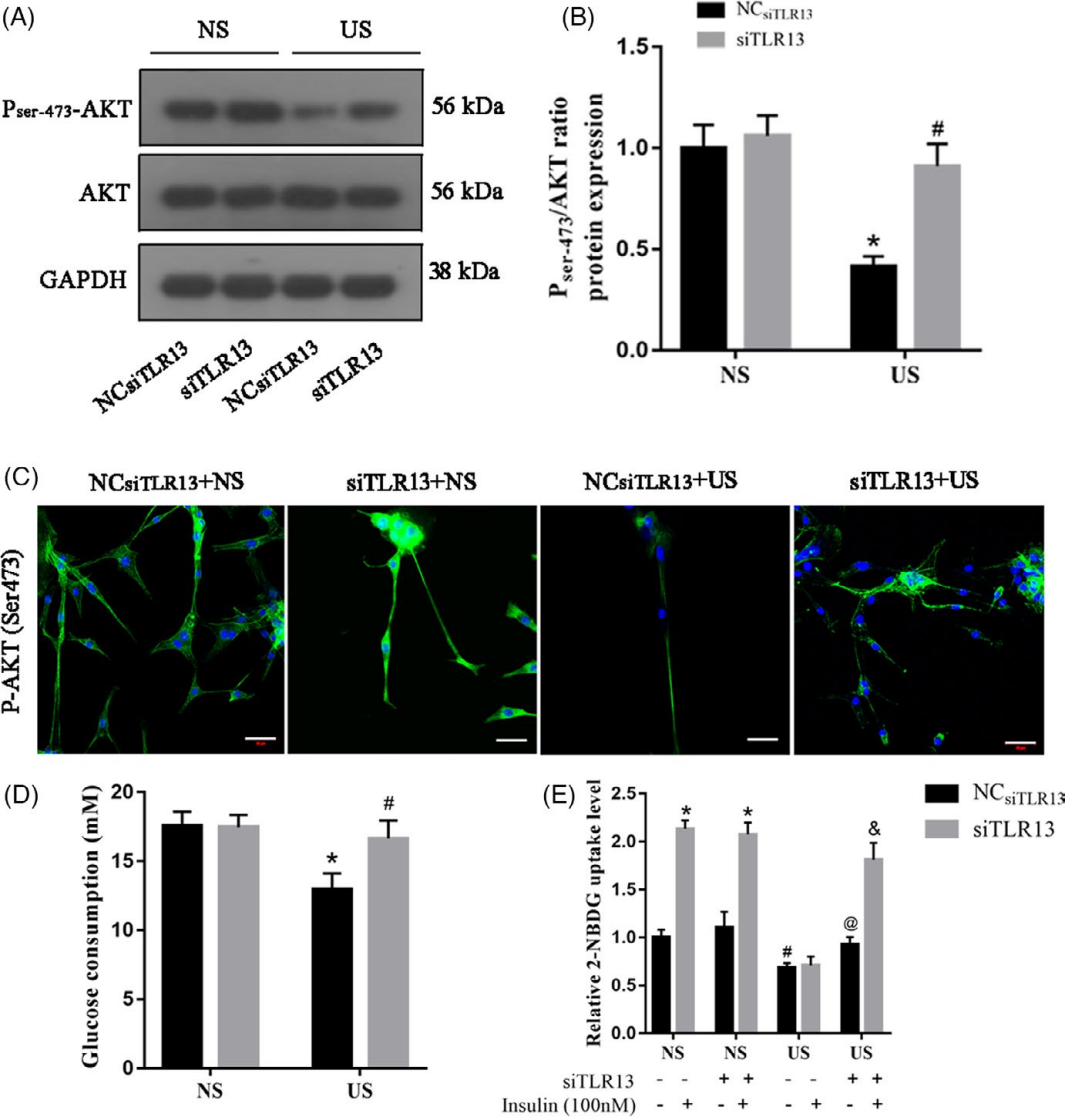
TLR13 impairs insulin signaling in uremic serum‐treated C2C12 myotubes. (A, B) AKT and p‐AKT were measured by western blot analysis (mean ± SEM; *n* = 3, *p < 0.05 vs. normal serum, NS; ^#^ *p* < 0.05 vs. NC_siTLR13_+ uremic serum, US). (C) Immunofluorescence staining of p‐AKT (Ser473) in C2C12 myotubes (scale bar = 50 μm). (D) Glucose consumption of C2C12 cells incubated with NS or US medium with or without knockdown of TLR13 (mean ± SEM; *n* = 6, *p < 0.05 vs. NS ^#^ *p* < 0.05 vs. NC_siTLR13_+US). (E) C2C12 cells were incubated under the above conditions in the absence (−) or presence (+) of insulin, and 2‐NBDG uptake was determined (mean ± SEM; *n* = 6, *p < 0.05 vs. their related basal group without insulin stimulation, ^#^ *p* < 0.05 vs. NS without insulin stimulation, ^@^ *p* < 0.05 vs. US without insulin stimulation, ^&^ *p* < 0.05 vs. US with insulin stimulation)

AKT regulates insulin resistance. Therefore, we examined the levels of phosphorylated AKT in C2C12 myotubes with and without the expression of TLR13. Western blotting analysis indicated that levels of p‐AKT at Ser‐473 were decreased under uremic serum compared with normal serum (Figure 4A,B). In immunofluorescent images (Figure 4C), uremic serum was found to inhibit p‐AKT (Ser‐473) in C2C12 myotubes. However, the silencing of TLR13 was able to increase the Ser‐473 phosphorylation of AKT under uremic serum. Glucose consumption in response to TLR13 knockdown was increased in C2C12 cells under uremic serum whereas consumption was reduced with TLR13 expressed (Figure 4D), indicating that TLR13 expression is associated with impaired insulin signaling. This was further confirmed in a 2‐NBDG uptake assay in the absence or presence of insulin (Figure 4E). Overall, our results suggest that TLR13 impairs insulin signaling and mediates insulin resistance in response to uremic serum in differentiated muscle cells.

### 3.5 TLR13 inhibition of AKT activity is dependent on IRF3 in muscle cells

**FIGURE 5:**
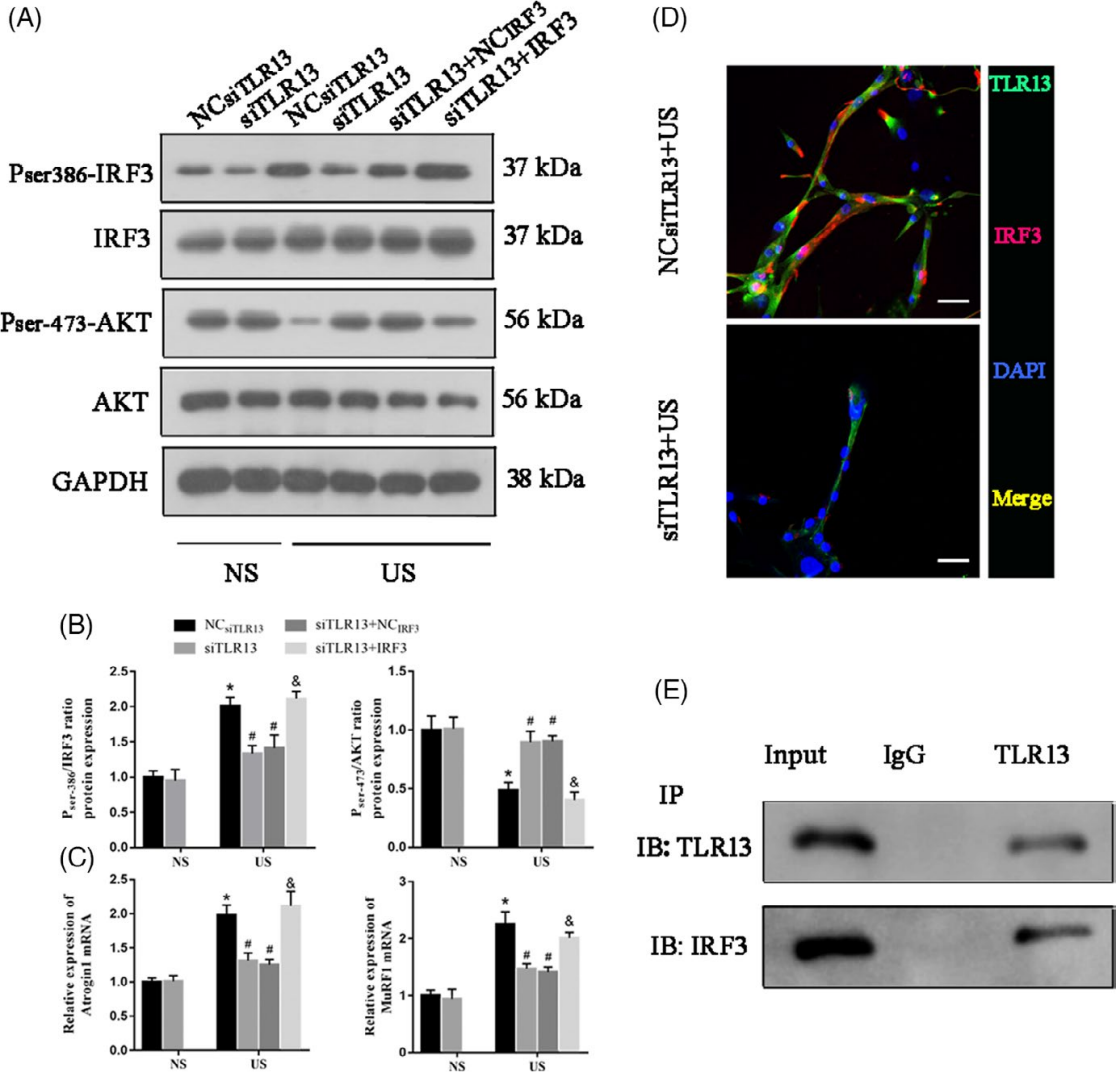
TLR13 regulates AKT activity via IRF3. (A, B) Western blot analysis of IRF3 and AKT activity in uremic serum (US)‐treated C2C12 myotubes transfected with TLR13 siRNA and IRF3 overexpression lentivirus (mean ± SEM; *n* = 3, *p < 0.05 vs. normal serum, NS; ^#^ *p* < 0.05 vs. NC_siTLR13_+US, ^&^ *p* < siTLR13+NC_IRF3_+US). (C) The mRNA level of muscle atrophic factors (atrogin‐1 and MuRF‐1) (mean ± SEM; *n* = 3, *p < 0.05 vs. normal serum, ^#^ *p* < 0.05 vs. NC_siTLR13_+US, ^&^ *p* < siTLR13+NC_IRF3_+US). (D) Immunofluorescence of P_ser386_‐IRF3 (green) and TLR13 (red) after TLR13 overexpression in US‐treated C2C12 myotubes (scale bar = 50 μm). (E) The expression of TLR13 and IRF3 was detected using Co‐IP

We discovered that TLR13 could interact with IRF3 in STRING predictive analysis. Therefore, we assessed whether IRF3 was also involved in the inhibition of AKT activity by measuring its phosphorylation in uremic serum‐treated C2C12 myotubes transfected with TLR13 siRNA and IRF3 overexpression lentivirus. In western blot analysis, the ratio of phosphorylated IRF3 (Ser‐386) was higher when TLR13 was expressed or IRF3 was overexpressed than when TLR13 was inhibited (Figure 5A,B). Correspondingly, the ratio of phosphorylated AKT (Ser‐473) was lower when *Tlr13* was expressed or when *Irf3* was overexpressed. As expected, levels of muscle atrophic factors (atrogin‐1 and MuRF‐1) were significantly higher when *Tlr13* was expressed or *Irf3* was overexpressed (Figure 5C). To confirm that the inhibition of AKT activity by TLR13 depends on IRF3, co‐localization of TLR13 and IRF3 expression was measured by immunofluorescence staining and coimmunoprecipitation (Co‐IP) assays (Figure 5D,E) and suggests that TLR13 combines with IRF3.

### 3.6 ETS2 activation stimulates *Tlr13* expression in C2C12 cells

**FIGURE 6:**
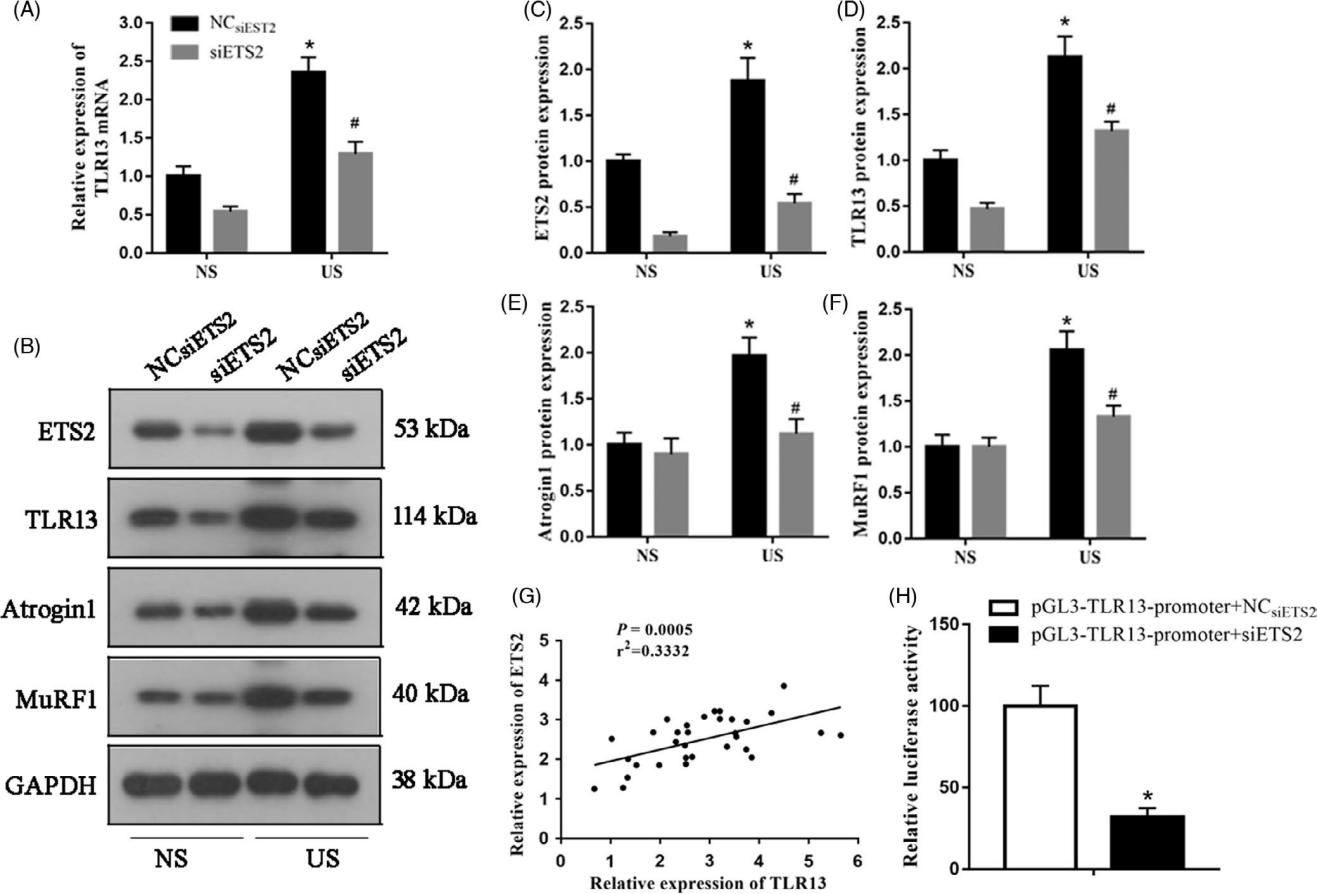
Transcription factor ETS2 could regulate TLR13 expression in C2C12 myotubes. (A) RT‐PCR analysis of ETS2 knockdown on TLR13 mRNA (mean ± SEM; *n* = 3, *p < 0.05 vs. normal serum, NS; ^#^ *p* < 0.05 vs. NC_siETS2_+ uremic serum, US). (B–F) Western blot analysis of TLR13 and muscle atrophic factors (atrogin‐1 and MuRF‐1) in NS or US‐treated C2C12 myotubes transfected with ETS2 siRNA (mean ± SEM; *n* = 3, *p < 0.05 vs. NS, ^#^ *p* < 0.05 vs. NC_siETS2_+US). (G) Correlation analyses indicated a positive linear association between the expression of ETS2 and TLR13. (H) Dual‐luciferase reporter gene assay to verify the regulatory relationship between EST2 and TLR13 (mean ± SEM; *n* = 6, *p < 0.05)

After establishing that IRF3 may be physically associated with TLR13, we next determined whether TLR13 may be regulated by the transcription factor ETS2. The relative mRNA expression and protein levels of TLR13 were reduced when ETS2 was silenced (Figure 6A–D). Correspondingly, levels of muscle atrophic factors were also lower when ETS2 was silenced (Figure 6E,F). Regression analysis indicated a positive correlation between ETS2 and TLR13 (Figure 6G), and a dual‐luciferase reporter gene assay confirmed a regulatory relationship (Figure 6H). These results show that ETS2 activation stimulates *Tlr13* expression.

### 3.7 TLR13 knockdown mice are protected from CKD‐induced skeletal muscle atrophy

**FIGURE 7:**
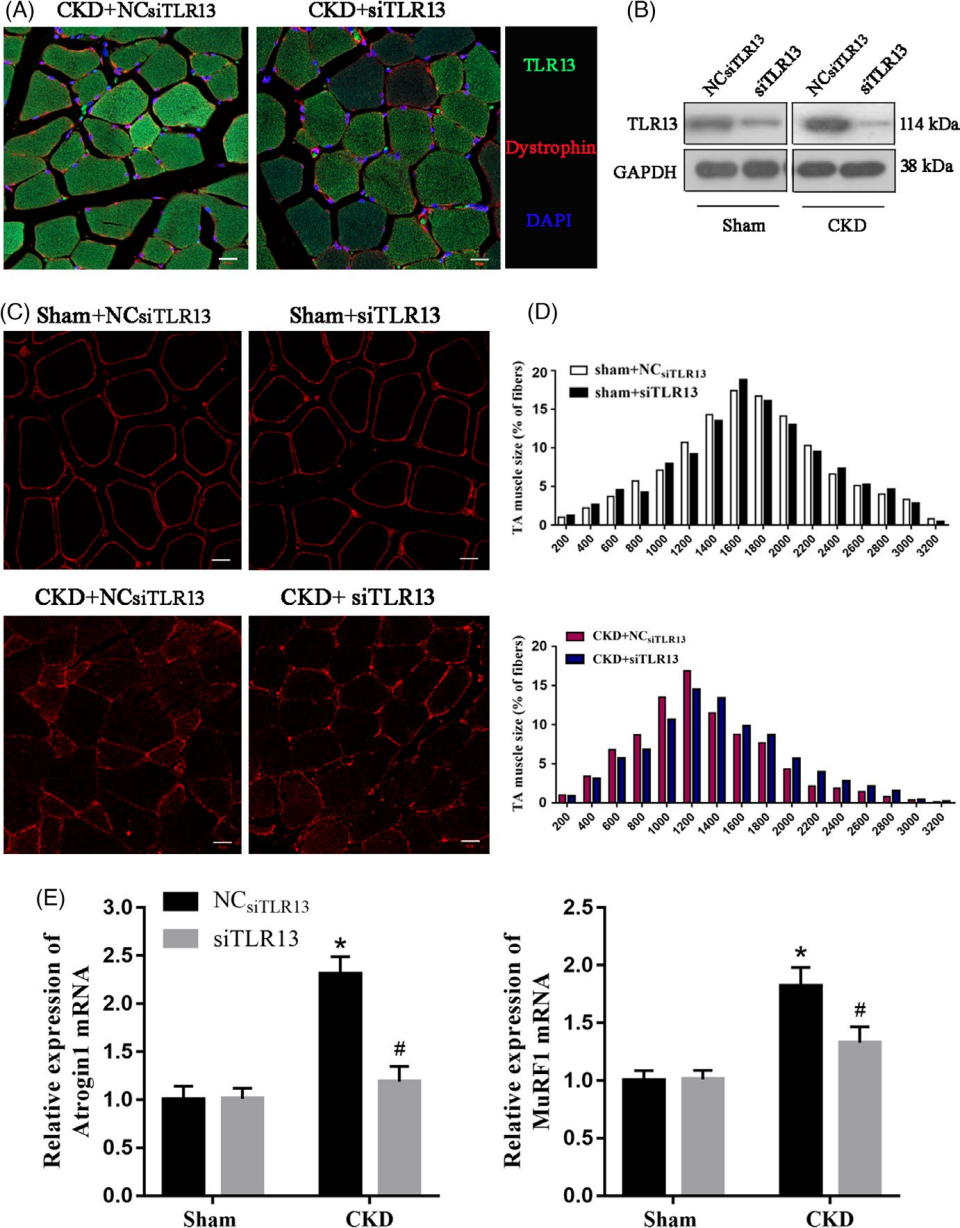
TLR13 knockdown alleviates chronic kidney disease (CKD)‐induced loss of muscle mass. (A) Tibialis anterior (TA) muscles of normal C57B6J mice were transfected by intramuscular injection of TLR13 siRNA. Cross‐sections of the tibialis anterior muscle from CKD versus control mice were immunostained with TLR13 (red) (scale bar = 20 μm). (B) Tibialis anterior (TA) muscles of control (CTL) and CKD mice were transfected with scrambled control siRNA (siCTL) or siRNA for TLR13 (siTLR13). The expression of TLR13 (mRNA and protein) was examined by immunoblots at 12 days after electroporation (mean ± SEM; *n* = 6). (C, D) The cross‐sectional area of myofibers in TA muscles of CTL and CKD mice following electroporation with siCTL and siTLR13 is shown. The distribution of myofiber sizes in muscles from sham‐operated mice treated with siCTL and siTLR13 was comparable (upper panel, data were obtained from three animals in each group). In the muscles of CKD mice treated with siTLR13, the distribution of myofiber sizes was shifted toward the right. Data were obtained from six animals in each group (scale bar = 20 μm). (E) The expression of atrogin‐1 and MuRF1 mRNAs was accessed by RT‐PCR. TLR13 knockdown suppressed atrogin‐1 and MuRF1 expression in the muscle of CKD mice (mean ± SEM; *n* = 6, *p < 0.05 vs. sham group, ^#^ *p* < 0.05 vs. NC_siTLR13_+CKD)

We investigated whether the results obtained in vitro could reverberate in an in vivo model of CKD in mice. TLR13 was inhibited in the TA muscles of mice by intramuscular injection of TLR13 siRNA. Immunofluorescent staining indicated that TLR13 was expressed at the highest level in the CKD mouse model injected with the negative control and at the lowest level in mice with *Tlr13* silenced (Figure 7A,B). The consequence of TLR13 knockdown in CKD mice was assessed by the distribution of myofiber sizes. In CKD mice, there was a significant decrease in the average cross‐sectional area of myofibers after transfection with control siRNA. In contrast, when TA muscles of CKD mice were transfected with TLR13 siRNA, the reduction in the average cross‐sectional area was significantly improved (Figure 7C). In addition, the distribution of myofibers in TA muscles shifted rightward when compared with results in CKD mice that had been electroporated with control siRNA (Figure 7D). The silencing of *Tlr13* increased the size of myofibers. In addition, muscle atrophic factors were upregulated in the AT muscle of the CKD model; however, the knockdown of *Tlr13* suppressed atrogin‐1 and MuRF1 expression in the muscle of CKD mice (Figure 7E). These results demonstrate that TLR13 knockdown protects mice from CKD‐induced loss of muscle mass.

### 3.8 TLR13 knockdown improves insulin resistance in vivo

**FIGURE 8:**
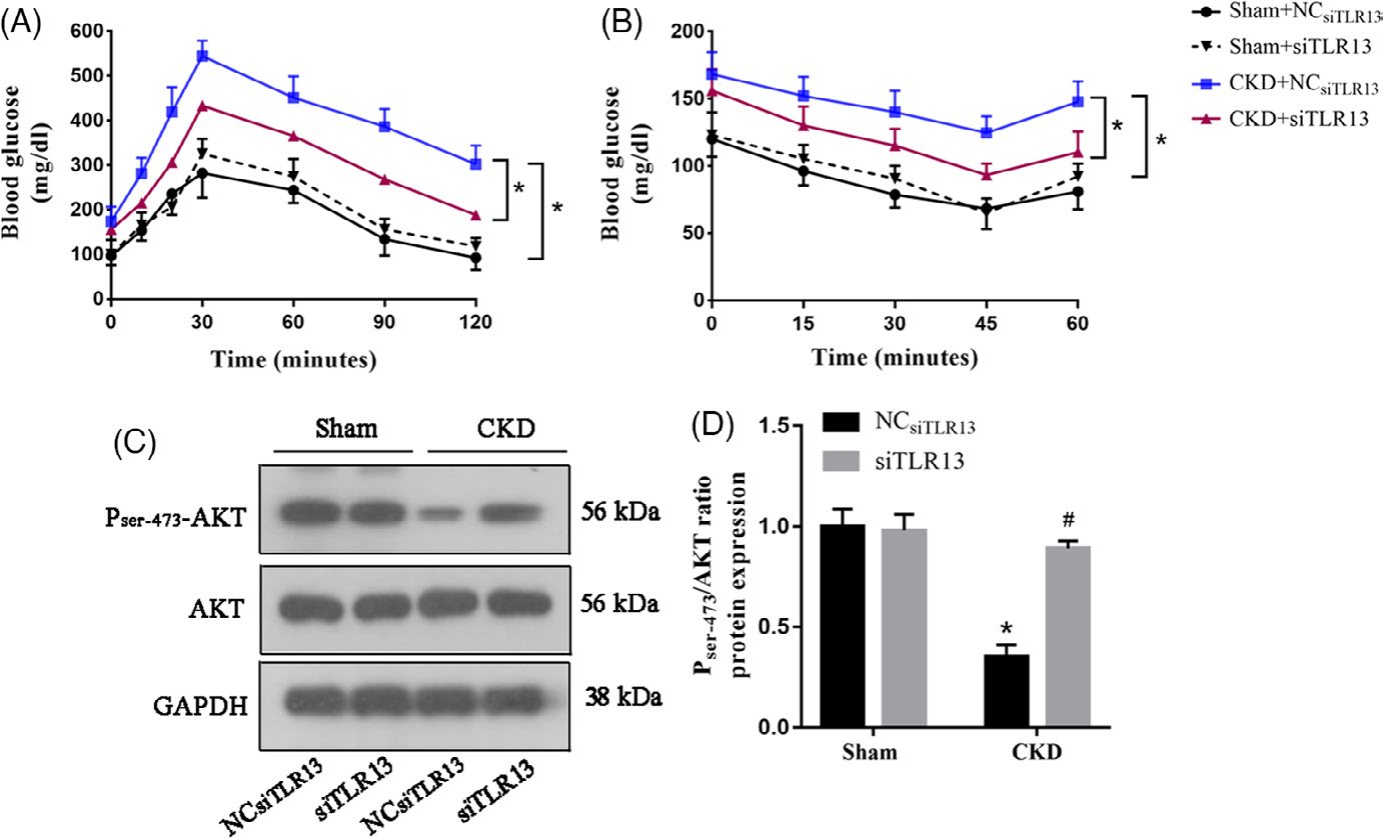
Knockdown of TLR13 reduces muscle TLR13 levels and improves glucose and insulin tolerance. (A) Intraperitoneal glucose tolerance test was performed in CKD versus control (CTL) mice after 16 h of fast (*p < 0.05, *n* = 6). (B) Intraperitoneal insulin tolerance test was performed in CKD versus CTL mice after a 4‐h fast (*p < 0.05, *n* = 3‐4). (C–D) Western blot analysis of AKT phosphorylation in tibialis anterior (TA) muscles in CKD versus CTL mice transfected with TLR13 siRNA

In individuals with insulin resistance, insulin‐stimulated glucose uptake is reduced significantly in skeletal muscle leading to the development of atrophy. Therefore, we performed glucose and insulin tolerance tests in the CKD mouse model with TLR13 knockdown. The knockdown of TLR13 reduced the levels of glucose in the CKD models (Figure 8A,B). Moreover, phosphorylation of AKT Ser‐473 was increased in the CKD model when TLR13 was suppressed (Figure 8C,D). Overall, these results suggest that TLR13 exacerbates muscle wasting in CKD and the knockdown of *Tlr13* improves glucose and insulin tolerance and ameliorates muscle atrophy.

## 4 DISCUSSION

The development of atrophy in skeletal muscle is associated with a reduction in insulin‐stimulated glucose uptake and protein degradation.^33^, ^34^ Therefore, we aimed to identify some of the downstream signaling elements responsible for the coordination of insulin control and protein degradation in skeletal muscle. We found that the murine proinflammatory factor TLR13 can recruit IRF3, leading to increased serine phosphorylation levels with decreased AKT phosphorylation. The latter stimulates protein degradation through the ubiquitin‐proteasome system.^35^ Our results suggest that this occurs by the following sequence of events: (1) *Ets2* is upregulated by CKD; (2) TLR13 levels are increased by the activation of ETS2; (3) TLR13 recruits IRF3 to increase its serine phosphorylation, thereby impairing intracellular AKT signaling; and (4) increased levels of TLR13 promote skeletal muscle atrophy.

The relationship between CKD and insulin resistance has been well documented.^36^ Obesity, diabetes mellitus, hypertension, and cardiovascular disease are believed to be among the underlying conditions that give rise to the low‐grade inflammation that impairs insulin signaling.^37^ Several studies have also noted an association between CKD‐induced insulin resistance and muscle protein wasting.^4^, ^38^ Protein degradation is increased with CKD‐associated insulin resistance, and therefore, it is difficult to manage protein intake in these patients, especially those on a low‐protein diet.^4^

Toll‐like receptors have been found to play an essential role in CKD‐induced skeletal muscle atrophy and insulin resistance.^39^, ^40^ Patients with insulin resistance have high levels of plasma free fatty acids (FFAs), which are ligands of the TLR4 pathway. Liang et al.^40^ found that increasing levels of FFAs in lean subjects was sufficient to upregulate TLR4 and inflammatory pathways. In addition, TLR2 and TLR4 are known to impair insulin signaling by activating proinflammatory cytokines.^13^, ^39^ However, little is known about the effects of TLR13 in CKD‐induced skeletal muscle atrophy although it was identified by microarray analysis as being differentially regulated in the muscles of mice with CKD.^17^ In agreement with this, we found that levels of TLR13 were higher in the TA muscles of a CKD mouse model than in a sham control. We also found that the suppression of TLR13 could increase the area of myotubes and reduce proteolysis in C2C12 cells challenged with uremic serum. Overall, our results indicate a pivotal role for TLR13 in muscle atrophy in mice, suggesting that it may act in a similar way to TLR2 and TLR4 in humans.

*Ets2* is not directly associated with muscle atrophy but is known to be an oncogene that drives various cancers through the aberrant activation of RAS/MAPK signaling, and it is known to interact with TLR13 and other TLRs.^18^, ^41^ In addition, ETS2 is associated with the regulation of inflammatory cytokines although there are contradictory reports about its regulation of IL‐6.^42^, ^43^ Cheng et al.^42^ found that ETS2 promotes an enhanced inflammatory state in epithelial cells that correlates with a higher level of IL‐6. However, in a more recent study, ETS2 was found to bind to the promoter of IL‐6 to inhibit transcription.^43^ We found that *Ets2* was upregulated in CKD and is associated with increased levels of TLR13. Moreover, levels of TLR13 mRNA and muscle atrophic factors were reduced when ETS2 was silenced. Therefore, we propose that the ETS2 protein may promote the expression of TLR13. Further studies are needed to identify the exact pathways involved.

The relationship between IRF3 and TLRs has been well documented with several studies reporting an interaction between TLR4/TRIF/IRF3 signaling.^44^, ^45^, ^46^ IRF3 has been implemented in the development of insulin resistance and suggested as a possible therapeutic target.^22^ The manipulation of TLRs that lay upstream of IRF3 was found to influence insulin resistance in murine adipocytes whereas the knockdown of IRF3 abolished insulin resistance. However, IRF3 is also associated with the alleviation of insulin resistance and the global knockdown of IRF3 has resulted in elevated levels of insulin resistance in mice.^47^ We found that TLR13 may be involved in the post‐translational modification of IRF3. TLR13 increases serine phosphorylation in IRF3 to impair intracellular insulin signaling. Thereby, increased levels of TLR13 will promote skeletal muscle atrophy. Targeting TLR13 may prove to be a therapeutic modality by improving insulin signaling and preventing the profound catabolic consequences of insulin resistance in CKD. Although this study suggests the involvement of TLR13 in skeletal muscle atrophy during CKD, the clinical relevance of blocking TLR13 to slow disease progression still needs to be evaluated.

AKT pathways have been associated with CKD‐induced skeletal muscle atrophy and insulin resistance.^48^, ^49^ In addition, AKT pathways have been associated with the suppression of IRF3 and the reduction of inflammation in models of hyperglycemia.^27^ In contrast, AKT pathways are also associated with the activation of IRF3 under hyperglycemia.^28^ We found that the TLR13 inhibition of the AKT pathway was dependent on IRF3 and that the ratio of phosphorylated AKT (Ser‐473) was lower when TLR13 was expressed or IRF3 was overexpressed. IRF3 is a key molecule in the induction of IFN‐β. IFN‐β induces insulin resistance by upregulating the expression of different SOCS isoforms in adipocytes.^50^ Deficiency in plasmacytoid dendritic cells and type I IFN signaling prevents diet‐induced obesity and insulin resistance in mice.^51^ There are also reports on the inhibitory effect of IFN‐β on p‐AKT. IFN‐β treatment has been shown to downregulate the PI3K/Akt pathway in neuroblastoma cells, and it is conceivable that IRF3‐induced p‐AKT inhibition is mediated through the IFN‐β/PI3K pathway.^52^ Nevertheless, how IRF3 inhibits the phosphorylation of Akt kinase in muscle during CKD remains to be investigated.

There are limitations to this study. We cannot exclude other factors that interact with TLR13 and contribute to CKD‐induced skeletal muscle atrophy. Further research will be needed to determine other genes and pathways that are influenced by the upregulation of TLR13 in CKD.

In conclusion, we have found that increased levels of TLR13 promote skeletal muscle atrophy and that TLR13 recruits IRF3, thereby impairing intracellular insulin signaling. The recruitment of IRF3 with TLR13 resulted in decreased AKT activity, whereas IRF3 knockdown prevented TLR13‐induced insulin resistance and increased p‐AKT. TLR13 is part of a novel mechanism of CKD‐mediated insulin resistance in muscle. Targeting the human equivalent to TLR13 or genes that suppress it may be a therapeutic modality in improving insulin signaling and preventing the profound catabolic consequences of insulin resistance in CKD.

## CONFLICT OF INTEREST

The authors declared no conflict of interest in this manuscript.

## AUTHOR CONTRIBUTIONS

SR and LJG participate in the processes of the conception and design of the study. LJG, ZFW, YYZ, NZ, JYL, and MY participate in the processes of experiment execution, data collection. ZFW, NZ, JYL, and LW participate in the processes of data analysis and interpretation of data. LJG, ZFW, YYZ, NZ, MY, and LW participate in the processes of manuscript preparation and the final manuscript improvement. All authors read and approved the final manuscript.

## ETHICS APPROVAL AND CONSENT TO PARTICIPATE

This study was approved by the Institutional Review Board of Shanghai General Hospital, Shanghai Jiao Tong University. All animal experiments were conducted in agreement with the Guide for the Care and Use of Laboratory Animals and were approved by the Committee of Shanghai General Hospital, Shanghai Jiao Tong University.

## Data Availability

The data that support the findings of this study are available from the corresponding author upon reasonable request.
